# Patterns of Perioperative Treatment and Survival of Localized, Resected, Intermediate- or High-Grade Soft Tissue Sarcoma: A 2000–2017 Netherlands Cancer Registry Database Analysis

**DOI:** 10.1155/2021/9976122

**Published:** 2021-07-22

**Authors:** Milan Van Meekeren, Marta Fiocco, Vincent K. Y. Ho, Judith V. M. G. Bovée, Hans Gelderblom, Rick L. Haas

**Affiliations:** ^1^Department of Medical Oncology, Leiden University Medical Center, Leiden, Netherlands; ^2^Department of Medical Statistics and Bioinformatics, Leiden University Medical Center, Leiden, Netherlands; ^3^Department of Research, Netherlands Comprehensive Cancer Organisation (IKNL), Utrecht, Netherlands; ^4^Department of Pathology, Leiden University Medical Center, Leiden, Netherlands; ^5^Department of Radiotherapy, The Netherlands Cancer Institute, Amsterdam, Netherlands; ^6^Department of Radiotherapy, Leiden University Medical Center, Leiden, Netherlands

## Abstract

**Background:**

Standard therapy for localized soft tissue sarcoma (STS) is wide, limb-sparing resection. For intermediate- or high-grade tumors, (neo)adjuvant therapies are frequently added to the treatment plan. In this study, data from a Dutch nationwide database are used to (1) assess whether perioperative management of STS follows ESMO guidelines, (2) characterize prognostic factors for overall survival (OS), and (3) assess the association between perioperative treatment and survival.

**Methods:**

All intermediate- or high-grade, localized STS cases, who have undergone surgery and diagnosed between 2000 and 2017, were identified in the Netherlands Cancer Registry (NCR) database. Variables with demographic, treatment, and survival data were obtained. Survival curves were estimated by Kaplan–Meier's method, and the effect of prognostic factors on OS was assessed in a multivariable Cox regression analysis.

**Results:**

A total of 4957 patients were identified. There were slightly more males (54.7%). Median age at diagnosis was 64 years, and 53.6% of the tumors were located in the extremities. Radiotherapy (RT) was administered to 2481 (50.1%) patients, and 252 (5.1%) patients were treated with perioperative systemic chemotherapy. The total use of perioperative RT did not significantly change in the last 20 years, but the timing followed clinical guidelines: preoperative RT increased significantly (2000–2008: 3.7%, 2009–2017: 22.3%; *p* < 0.001), whereas the use of postoperative RT diminished (2000–2008: 45.9%, 2009–2017: 26.1%; *p* < 0.001). The use of perioperative chemotherapy slightly decreased (2000–2008: 5.9%, 2009–2017: 4.4%; *p* = 0.015). 5-year OS was 59.6% (95% CI: 58.2–61.0). Sex, age, year of diagnosis, tumor location, tumor size, histological grade, depth, histological subtype, surgical margins, and the use of perioperative RT were identified as independent predictors for OS.

**Conclusion:**

Preoperative RT is gradually replacing postoperative RT for localized STS in the Netherlands. The use of perioperative chemotherapy is rare and has slightly decreased in recent years. Identified baseline characteristics and treatment factors predicting OS may aid in future treatment decisions.

## 1. Introduction

Soft tissue sarcomas (STS) comprise a group of rare neoplasms that can arise in tissues of mesenchymal origin virtually anywhere in the body. They represent a heterogeneous group, with the WHO distinguishing over 80 histological subtypes [1]. Due to this heterogeneity and low incidence, finding optimal treatment strategies has been a challenge over the years. Surgery remains the most important treatment modality for localized STS, with wide, function-sparing resection being the primary objective [2–4]. (Neo)adjuvant radiotherapy should be considered for intermediate- and high-grade STS. Surgery alone is generally reserved for patients with small (<5 cm), superficial, and low-grade tumors [3]. High local control rates [5] are observed after the combination of radiotherapy (RT) and surgery. However, a substantial proportion of patients still develops distant metastases and eventually succumbs to their disease [6]. Therefore, multiple studies have been performed on (neo)adjuvant systemic therapies for localized STS, with the aim to reduce distant recurrences and improve patient survival. The role of perioperative chemotherapy for resectable STS remains controversial. The latest meta-analysis of all available randomized evidence on chemotherapy was published in 2008 [7], including 18 trials. Doxorubicin-based chemotherapy led to improved local-, distant-, and overall recurrence, while no improvement in overall survival was identified for doxorubicin alone. The combination of doxorubicin and ifosfamide on the other hand showed a statistically significant, but small overall survival improvement over treatment with no chemotherapy. Nonetheless, these benefits must always be weighed against the additional toxicities associated with chemotherapy. Over the past decade, targeted therapies have been introduced into cancer management. While early evidence suggests a role for these new biologicals in resectable STS as neoadjuvant treatment in combination with RT [8–11], further research is imperative to be able to draw definitive conclusions regarding their safety and efficacy. Based on the available literature, guidelines on perioperative treatment of STS are continuously updated. The latest version of the Dutch STS guidelines dates back to 2011 [12]. Since then, the biannual ESMO STS guidelines are the leading guidelines in the Netherlands [3]. Whether developments in these clinical guidelines have actually resulted in implementation into the clinic as well as in significant changes in outcomes for patients with this rare type of cancer in the Netherlands remains unclear. Therefore, in this study, data from the Netherlands Cancer Registry (NCR) have been used to describe the evolution of perioperative therapy for resected, intermediate- or high-grade STS in the Netherlands from 2000 until 2017. On the basis of this nationwide aggregated cancer patient dataset, robust characterization of overall survival (OS) and prognostic factors can be provided, which are additional aims of this study.

## 2. Methods

### 2.1. Data Source

In the Netherlands, there are five dedicated centres with specific expertise in sarcoma. However, a substantial number of patients is still being treated in peripheral hospitals. Aggregated patient data from all Dutch hospitals treating sarcoma patients registered in the nationwide Netherlands Cancer Registry (NCR) were used. Inclusion criteria for our study were all patients diagnosed with an intermediate- or high-grade and nonmetastasized STS between 2000 and 2017. For most tumors, grade was based on the FNCLCC grading system. For other tumors, older grading systems or data from the pathology-reports were used to determine the grade. Tumors that had unknown grade and were of undifferentiated subtype were deemed as grade III tumors and included in this study. Exclusion criteria were retroperitoneal, intra-abdominal, and gynaecological STSs and all patients not undergoing surgery.

### 2.2. Variables

Demographic data, treatment data, and survival data were obtained. Demographic data consisted of sex, year and age at diagnosis, tumor location, histological subtype, histological grade, and staging information. All tumors were subtyped according to the WHO 2013 classification [13], and not by the newer WHO 2020 classification [1], given the time of data capture. For the logistic regression and Cox regression analyses, liposarcomas were subdivided into myxoid liposarcoma, dedifferentiated liposarcoma, pleomorphic liposarcoma, and liposarcoma NOS because of their distinct clinical behaviour [14]. The age groups “young”, “old,” and “middle” represent evenly sized cohorts based upon age at diagnosis. Tumor size was extracted from the clinical T-stadium and the extent of disease-score, and tumor depth was extracted from the pathological T-stadium and/or clinical T-stadium. Subsequently, those tumors with unknown tumor depth on the basis of their T-stadium that were located in the head and neck region, heart, mediastinum, pleura, peripheral nerves, male genitals (others), or the thyroid gland were deemed as having a deep tumor depth. Those tumors located in the skin, breast, female external genitalia, or scrotum were deemed as having a superficial depth. Treatment data comprised a variable radicality of the surgery and radicality of a potential resurgery. If a patient had a resurgery, the radicality of this last surgery was used in the analyses. Other treatment variables were perioperative radiotherapy, systemic chemotherapy, and targeted therapy. Regarding survival data, duration of follow-up in days, both from the date of diagnosis and from the date of surgery and patient status (alive/dead) were analysed, yielding overall survival information. Unfortunately, local control data are not captured in the NCR.

### 2.3. Statistical Analysis

All statistical analyses were performed with IBM SPSS Statistics 25. Descriptive statistics were employed to describe baseline characteristics. Median follow-up was estimated with the reverse Kaplan–Meier method [15]. To test for treatment changes over time, chi-square tests were performed. To investigate the effect of baseline factors on the chance of receiving perioperative RT, univariate logistic regression models were estimated. The Kaplan–Meier method was used to estimate OS curves. Log-rank tests were utilized to assess differences between survival curves. Multiple imputations were used for five variables with missing values (WHO 2013 subtype, tumor grade, tumor size, tumor depth, and radicality of the surgery). For each imputed dataset, a Cox model was estimated. The final estimates were pooled with the Rubin's rule [16]. There was no violation of the proportional hazard assumption for each prognostic factor, evaluated by visual inspection of log-log survival. *p* < 0.05 was considered statistically significant.

## 3. Results

### 3.1. Demographics: Baseline Characteristics

The cohort consisted of 4957 patients, with slightly more males (54.7%, 2711 patients). Median age at diagnosis was 64 years (IQR 49–76 years). Most tumors (53.6%) occurred in the extremities, with the lower extremity being the most predominant site (39.4%). There were more high grade than intermediate grade tumors (65.5% vs. 34.5%, respectively). Most tumors were larger than 5 cm (44.5% vs. 33.4%, respectively), and most were located superficially (51.3% vs. 33.4%, respectively). More than half of the patients underwent R0 surgery (56.5%), and 15.8% had positive surgery margins (R1/R2).  Table 1 presents an overview of baseline characteristics.

### 3.2. Demographics: Histological Subtype

**Figure 1:**
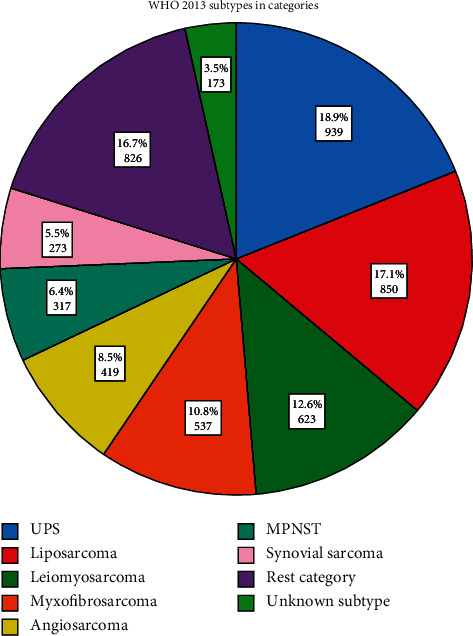
Overview of histological subtypes of grade II and III resected soft tissue sarcoma in the Netherlands between 2000 and 2017. Abbreviations: UPS = undifferentiated pleomorphic sarcoma, MPNST = malignant peripheral nerve sheath tumor.

All tumors are presented with their respective histological subtype according to the WHO 2013 classification (Figure 1). Undifferentiated pleiomorphic sarcoma (UPS) was the most common subtype in this cohort (18.9%), followed by liposarcoma (17.1%) and leiomyosarcoma (12.6%).

### 3.3. Adjuvant Treatment

**Figure 2:**
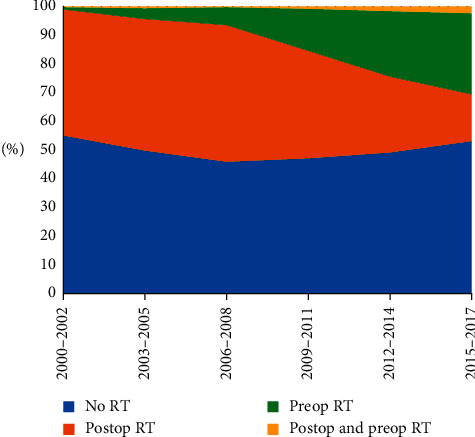
The use of perioperative radiotherapy for grades II and III resected soft tissue sarcoma in the Netherlands between 2000 and 2017. Abbreviations: RT = radiotherapy, postop = postoperative, and preop = preoperative.

A total of 2481 (50.1%) patients received radiotherapy. 13.8% of all patients were radiated preoperatively, 35.1% postoperatively, and 1.1% both pre- and postoperatively. In Figure 2 and Table 2, an overview of RT use over time is given. No statistically significant change in the overall use of RT was observed in the second half of this study period versus the first half (2000–2008: 50.1%, 2009–2017: 50.0%; *p*=0.984). However, preoperative RT showed a statistically significant increase (2000–2008: 3.7%, 2009–2017: 22.3%; *p* < 0.001), whereas the use of postoperative RT diminished (2000–2008: 45.9%, 2009–2017: 26.1%; *p* < 0.001).

**Figure 3:**
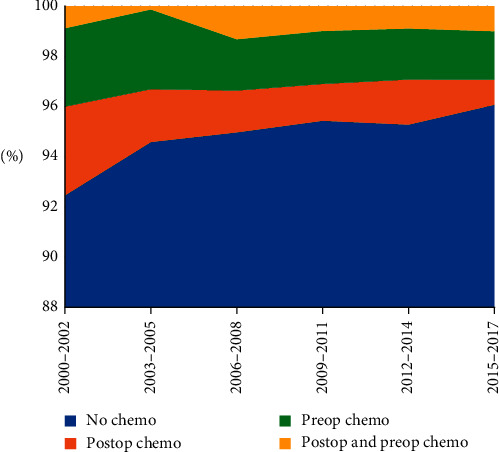
The use of perioperative chemotherapy for grades II and III resected soft tissue sarcoma in the Netherlands between 2000 and 2017. Abbreviations: chemo = chemotherapy, postop = postoperative, and preop = preoperative.

A total of 252 (5.1%) patients were treated with adjuvant systemic chemotherapy, 116 preoperatively (2.3%), 92 postoperatively (1.9%), and 44 pre- and postoperatively (0.9%).  Figure 3 and Table 3 show that, overall, the use of systemic chemotherapy decreased over time, from 5.9% in 2000–2008 to 4.4% in 2009–2017 (chi-square test: *p*=0.015).

The results of the univariable logistic regression analysis on the chance of receiving (neo)adjuvant RT are shown in Table 4.

### 3.4. Overall Survival

Follow-up data were available for 4923 out of 4957 patients (Table 5).

Figures S1–S12 show survival curves for different risk factors. The equally sized age group curves, subtype curves, tumor location curves, tumor grade curves, tumor size curves, tumor depth curves, radicality of the surgery curves, and perioperative RT all differ significantly from each other, compared by log rank tests (*p* < 0.001).  Tables S1–S11 report on corresponding OS rates.

In Table 6, an overview of prognostic factors for OS, corrected for the other variables in the model, is presented.

## 4. Discussion

This study shows that approximately half (50.1%) of grades II and III STS patients treated with surgery between 2000 and 2017 received perioperative RT in the Netherlands. In 2002, the SR2-trial was published [17–19], which showed that preoperative and postoperative radiation have comparable local control rates and survival. However, patients in the preoperative arm of the trial experienced a significantly lower incidence of late, often irreversible morbidities, albeit at the cost of a higher rate of acute wound complications. Earlier ESMO STS guidelines, up until 2012 [20–25], and the Dutch national guideline for management of STS of 2004 [26] all state a preference for the postoperative timing of RT. In the 2014 [27] and 2018 [3] ESMO guidelines, a preference for preoperative RT becomes apparent, with the recommendation to use preoperative radiation for those patients for which acute wound problems are expected to be a manageable problem. The last Dutch national STS guideline of 2011 follows this shift towards recommending preoperative radiation [12]. These guideline revisions are reflected in our study, which showed that preoperative RT for grade II and III STS was used significantly more from 2009–2017 than from 2000–2008, whereas the use of postoperative RT diminished significantly in this period. The latest ESMO guideline [3] suggests that perioperative RT is the standard treatment for intermediate/high grade, >5 cm, deep STS. Earlier ESMO guidelines also recommend perioperative RT for high-risk sarcomas. In our study, which only included grade II and grade III sarcomas, RT was used in only half of the patients. The results from the univariable logistic regression analysis show that patients with a high-grade sarcoma, tumor size >5 cm, or deeply located sarcoma more often received perioperative RT. Tumor size >5 cm showed the strongest association with perioperative RT (OR 2.418, 95% CI: 2.122–2.756). In our analysis, age is also a predictor for receiving radiotherapy, with each additional life-year at diagnosis significantly decreasing the chance of receiving radiotherapy by 1.8% (95% CI 1.4–2.1). As shown in Table 4, male patients had a higher chance of being radiated perioperatively than female patients. With respect to tumors located in the lower extremity, other STSs had a significantly lower chance of receiving perioperative radiation. Myxofibrosarcomas and synovial sarcomas are reported to have an increased risk of local recurrence after surgery relative to other histological subtypes [28, 29], and synovial sarcomas were historically considered as high-grade tumors, which might be an explanation for the above average use of perioperative RT for these subtypes. Myxoid liposarcomas (MLS) are known to have a marked radiosensitivity [30], which possibly explains the high number of MLS patients in our cohort receiving perioperative RT.

The use of (neo)adjuvant chemotherapy for localized STS is still under debate. In our cohort, perioperative chemotherapy was significantly less prescribed to patients with grades II or III STS from 2009 to 2017 (4.4%), than it was from 2000 to 2008 (5.9%). The opinions differ on whether the marginal survival benefits found for combination chemotherapy in the 2008 meta-analysis mean that chemotherapy should be implemented into the standard of care for STS. No STS guideline has taken up chemotherapy as standard therapy [3, 12]. A 2017 survey [31], for which EORTC medical oncology experts were asked about their center's policies on (neo)adjuvant chemotherapy for STS, showed that, in line with the preoperative shift in radiotherapy, the interest in neoadjuvant systemic therapies has also risen. Neoadjuvant treatment of sarcomas has potential benefits of allowing more conservative surgeries, in addition to earlier treatment of possible micrometastases. Finally, with the tumor still in situ, there is a unique opportunity of histotype-tailored radiological and pathological treatment response evaluation to adjust individual treatment accordingly. The addition of (neo)adjuvant radiotherapy to surgery has the potential of increasing the local control probability. The addition of radiosensitizers may further intensify the management, intending to decrease local recurrence rates and possibly even long-term radiation-associated side effects [32, 33]. Future investigations should focus on identifying individual patients or subtypes that might benefit from (neo)adjuvant systemic chemotherapy. Synovial sarcoma, according to some reports, may have a relatively high chemosensitivity [29]. Patients with this subtype were most frequently treated with chemotherapy in our cohort (Figure S13), and chemotherapy use for angiosarcoma has markedly increased in recent years.

Prescription of perioperative targeted therapy in this dataset was first observed in 2007 (Table S12). Haas et al. suggested that the combination of neoadjuvant RT and pazopanib for localized STS is tolerable and has promising antitumor efficacy [11]. These radiosensitizing efforts hold great promise for the future and are expected to be extensively studied in coming years.

In recent years, tools have been developed for the prediction of OS on the basis of certain prognostic factors. Examples of such prediction tools are SARCULATOR [6] and PERSARC [34]. Our study showed that, for two patients that were exactly the same regarding the other variables in the Cox regression model, but one was diagnosed in the second half of the study (2009–2017) and the other one in the first half (2000–2008), the latter had an approximately 17% higher chance of dying (HR: 1.169; 95% CI: 1.072–1.274). Age, sex, tumor location, tumor grade, tumor size, tumor depth, and resection margins were also associated with survival. All of these prognostic factors were identified by the PERSARC and SARCULATOR models. For assessing the impact of certain histological subtypes on OS, UPS was our reference subtype. Leiomyosarcoma (HR: 1.228, 95% CI: 1.060–1.422), angiosarcoma (HR: 1.631, 95% CI: 1.338–1.988), and MPNST (HR: 1.328, 95% CI: 1.079–1.635) showed significantly worse survival, myxoid liposarcoma (HR: 0.613, 95% CI: 0.480–0.783) and dedifferentiated liposarcoma (HR: 0.805, 95% CI: 0.652–0.995) had significantly better survival, and no survival difference was observed between UPS and myxofibrosarcoma, synovial sarcoma, pleomorphic liposarcoma, liposarcoma NOS, or the rest category. These results might contribute to more extensive, personalized prediction tools in the future to more accurately identify patients at a higher risk of dying, so more aggressive treatments can be considered for this subset of patients. This study suggests an association between perioperative RT and overall survival (RT yes vs. RT no; HR: 0.810; 95% CI: 0.741–0.886), which has been reported in other retrospective soft-tissue sarcoma database studies [35, 36]. An association between perioperative chemotherapy and overall survival was not found. Our study has several limitations. First, the survival benefit by the addition of RT should be interpreted with caution. A robust statement of a causative effect of a certain treatment should, obviously, be obtained by randomized clinical trials. Although the Cox regression model provides insight into the effect of adjuvant treatments on survival, corrected for the other variables in the model, these results cannot directly be translated into clinical recommendations and guidelines. Second, our study is restricted to data that is registered by the NCR. The NCR only captures date of death, so we were able to report OS. Because no data regarding cause of death, metastases, or local recurrences are registered in the database, oncologic outcomes like disease-specific survival (DSS) or metastasis-free survival (MFS) could not be reported. Furthermore, information about local tumor control is not available in the NCR.

## 5. Conclusions

This study showed treatment patterns for resectable nonmetastatic intermediate and high-grade STS almost over the last 2 decades. Although still relatively infrequently applied, the rate of preoperative RT is gradually increasing over the years in the Netherlands, which followed clinical guideline recommendations. Future STS research should focus on identifying prognostic factors and biomarkers on an individual patient basis to give doctors the means to tailor their treatments accordingly, thus improving patient survival chances and quality of life.

## Figures and Tables

**Table 1:** Patient characteristics for grade II and III resected soft tissue sarcoma in the Netherlands between 2000 and 2017.

Characteristics	
Total no. of patients	4957
Median follow-up (years)	10.0, 95% CI 9.6–10.4
*Sex*	
Male	2711 (54.7%)
Female	2246 (45.3%)

*Age (years)*	
<40	689 (13.9%)
40–49	563 (11.4%)
50–59	808 (16.3%)
60–69	1020 (20.6%)
≥70	1877 (37.9%)

*Tumor location*	
Lower extremity	1954 (39.4%)
Upper extremity	705 (14.2%)
Trunk	1529 (30.8%)
Head and neck	727 (14.7%)
Heart/mediastinum/pleura	42 (0.8%)

*Grade*	
Intermediate (II)	1712 (34.5%)
High (III)	3245 (65.5%)^*∗*^

*Tumor size*	
≤5 cm	1654 (33.4%)
>5 cm	2208 (44.5%)
Size unknown/missing	1095 (22.1%)

*Tumor depth*	
Superficial	2542 (51.3%)
Deep	1655 (33.4%)
Depth unknown/missing	760 (15.3%)

*Radicality of the surgery*	
R0	2800 (56.5%)
R1/R2	785 (15.8%)
Radicality unknown/missing	1372 (27.7%)

^*∗*^606 tumors with unknown grade and of undifferentiated subtype were regraded as grade III tumors.

**Table 2:** The use of perioperative radiotherapy for grades II and III resected soft tissue sarcoma in the Netherlands between 2000 and 2017.

	No RT	Postop RT	Preop RT	Postop and preop RT
2000–2002	372 (55.0%)	297 (43.9%)	5 (0.7%)	2 (0.3%)
2003–2005	377 (49.8%)	347 (45.8%)	28 (3.7%)	5 (0.7%)
2006–2008	383 (45.9%)	397 (47.6%)	52 (6.2%)	2 (0.2%)
2009–2011	381 (47.1%)	303 (37.5%)	118 (14.6%)	7 (0.9%)
2012–2014	437 (49.1%)	236 (26.5%)	202 (22.7%)	15 (1.7%)
2015–2017	526 (53.1%)	162 (16.3%)	279 (28.2%)	24 (2.4%)

Abbreviations: RT = radiotherapy, postop = postoperative, preop = preoperative.

**Table 3:** The use of perioperative chemotherapy for grades II and III resected soft tissue sarcoma in the Netherlands between 2000 and 2017.

	No chemo	Postop chemo	Preop chemo	Postop and preop chemo
2000–2002	625 (92.5%)	24 (3.6%)	21 (3.1%)	6 (0.9%)
2003–2005	716 (94.6%)	16 (2.1%)	24 (3.2%)	1 (0.1%)
2006–2008	792 (95.0%)	14 (1.7%)	17 (2.0%)	11 (1.3%)
2009–2011	772 (95.4%)	12 (1.5%)	17 (2.1%)	8 (1.0%)
2012–2014	848 (95.3%)	16 (1.8%)	18 (2.0%)	8 (0.9%)
2015–2017	952 (96.1%)	10 (1.0%)	19 (1.9%)	10 (1.0%)

Abbreviations: chemo = chemotherapy, postop = postoperative, and preop = preoperative.

**Table 4:** Estimated odds ratio (OR) along with 95% confidence interval (CI) estimated from univariable logistic regression models on the association between patient and tumor factors and the chance of receiving perioperative radiotherapy for grade II and III resected soft tissue sarcoma in the Netherlands between 2000 and 2017.

Factor	OR	95% CI	*p* value
Age (continuous)	0.982	0.979–0.986	<0.001^*∗*^
*Sex*			0.030^*∗*^
Female sex (ref.)	—	—	
Male sex	1.132	1.012–1.266	

*Location*			<0.001^*∗*^
Lower extremity (ref.)	—	—	
Upper extremity	0.769	0.645–0.916	
Head & neck	0.231	0.192–0.278	
Trunk	0.376	0.328–0.431	
Heart/mediastinum/pleura	0.255	0.132–0.494	

*Subtype*			<0.001^*∗*^
UPS (ref.)	—	—	
Myxofibrosarcoma	2.346	1.878–2.929	
Leiomyosarcoma	0.754	0.615–0.926	
Angiosarcoma	0.283	0.216–0.371	
MPNST	1.062	0.823–1.370	
Synovial sarcoma	1.866	1.415–2.462	
MLS	2.665	2.087–3.404	
Pleomorphic liposarcoma	1.827	1.249–2.671	
Dedifferentiated liposarcoma	0.857	0.638–1.151	
Liposarcomas NOS	1.370	0.846–2.219	
Rest category	0.885	0.734–1.068	

*Grade*			0.009^*∗*^
Intermediate grade II (ref.)	—	—	
High grade III	1.170	1.040–1.315	

*Tumor size*			<0.001^*∗*^
≤5 cm (ref.)	—	—	
>5 cm	2.418	2.122–2.756	

*Tumor depth*			<0.001^*∗*^
Superficial depth (ref.)	—	—	
Deep depth	1.660	1.465–1.880	

Abbreviations: OR=odds ratio, CI=confidence interval, ref. = reference, UPS = undifferentiated pleomorphic sarcoma, MPNST = malignant peripheral nerve sheath tumor, MLS = myxoid liposarcoma, NOS = not otherwise specified. ^*∗*^*p* < 0.05.

**Table 5:** OS at 1, 2, 5, and 10 years along with 95% confidence interval for grades II and III resected soft tissue sarcoma in the Netherlands between 2000 and 2017.

1-year OS	2-year OS	5-year OS	10-year OS
89.0% (88.2–89.8)	77.7% (76.5–78.9)	59.6% (58.2–61.0)	46.3% (44.7–47.9)

**Table 6:** Estimated hazard ratio (HR) along with 95% confidence interval from a multivariable Cox regression model on the association between prognostic factors and overall survival for grades II and III resected STS in the Netherlands between 2000 and 2017.

Factor	HR	95% CI	*p* value
*Age*			
Young (ref.)	—	—	—
Middle	1.651	1.471–1.854	<0.001^*∗*^
Old	3.323	2.952–3.740	<0.001^*∗*^

*Sex*			
Female sex (ref.)	—	—	—
Male sex	1.097	1.009–1.193	0.030^*∗*^

*Year of diagnosis*			
2009–2017 (ref.)	—	—	—
2000–2008	1.169	1.072–1.274	<0.001^*∗*^

*Location*			
Lower extremity (ref.)	—	—	—
Upper extremity	0.922	0.807–1.054	0.234
Head and neck	1.228	1.074–1.404	0.003^*∗*^
Trunk	1.183	1.066–1.314	0.002^*∗*^
Heart/mediastinum/pleura	2.177	1.464–3.235	<0.001^*∗*^

*Subtype*			
UPS (ref.)	—	—	—
Myxofibrosarcoma	0.885	0.747–1.048	0.157
Leiomyosarcoma	1.228	1.060–1.422	0.006^*∗*^
Angiosarcoma	1.631	1.338–1.988	<0.001^*∗*^
MPNST	1.328	1.079–1.635	0.008^*∗*^
Synovial sarcoma	0.986	0.797–1.219	0.895
MLS	0.613	0.480–0.783	<0.001^*∗*^
Pleomorphic liposarcoma	0.813	0.623–1.060	0.126
Dedifferentiated liposarcoma	0.805	0.652–0.995	0.045^*∗*^
Liposarcoma NOS	0.864	0.630–1.185	0.364
Rest category	0.968	0.839–1.116	0.651

*Grade*			
Intermediate grade II (ref.)	—	—	—
High grade III	1.417	1.264–1.589	<0.001^*∗*^

*Tumor size*			
≤5 cm (ref.)	—	—	—
>5 cm	1.631	1.451–1.833	<0.001^*∗*^

*Tumor depth*			
Superficial depth (ref.)	—	—	—
Deep depth	1.234	1.122–1.356	<0.001^*∗*^

*Perioperative RT*			
No (ref.)	—	—	—
Yes	0.810	0.741–0.886	<0.001^*∗*^

*Perioperative chemotherapy*			
No (ref.)	—	—	—
Yes	1.137	0.936–1.381	0.196

*Surgical margins*			
R0 margins (ref.)	—	—	—
R1/R2 margins	1.492	1.327–1.677	<0.001^*∗*^

Abbreviations: HR=hazard ratio, CI=confidence interval, ref. = reference, UPS = undifferentiated pleomorphic sarcoma, MPNST = malignant peripheral nerve sheath tumor, MLS = myxoid liposarcoma, NOS = not otherwise specified, RT = radiotherapy. ^*∗*^*p* < 0.05.

## Data Availability

The data used to support the findings of this study are available from the corresponding author upon request.

## Supplementary Materials

Supplementary Materials Figures S1–S12 show Kaplan–Meier survival curves, by the following risk factors: equally sized age groups, sex, year of diagnosis, subtype, tumor location, tumor grade, tumor size, tumor depth, radicality of the surgery, perioperative radiotherapy, and perioperative chemotherapy. Corresponding overall survival rates are provided in Tables S1–S11. Figure S13 shows the percentage of patients receiving perioperative chemotherapy per subtype. Table S12 shows the use of perioperative targeted therapy over the years.
