# Supplementary material for: Patterns of Perioperative Treatment and Survival of Localized, Resected, Intermediate- or High-Grade Soft Tissue Sarcoma: A 2000–2017 Netherlands Cancer Registry Database Analysis
Source: Sarcoma. 2021 Jul 22;2021:9976122. doi: 10.1155/2021/9976122 (PMC8324372; doi:10.1155/2021/9976122)
Supplement: Supplementary Materials — Figures S1–S12 show Kaplan–Meier survival curves, by the following risk factors: equally sized age groups, sex, year of diagnosis, subtype, tumor location, tumor grade, tumor size, tumor depth, radicality of the surgery, perioperative radiotherapy, and perioperative chemotherapy. Corresponding overall survival rates are provided in Tables S1–S11. Figure S13 shows the percentage of patients receiving perioperative chemotherapy per subtype. Table S12 shows the use of perioperative targeted therapy over the years. [file 9976122.f1.docx]

## Supplementary Materials


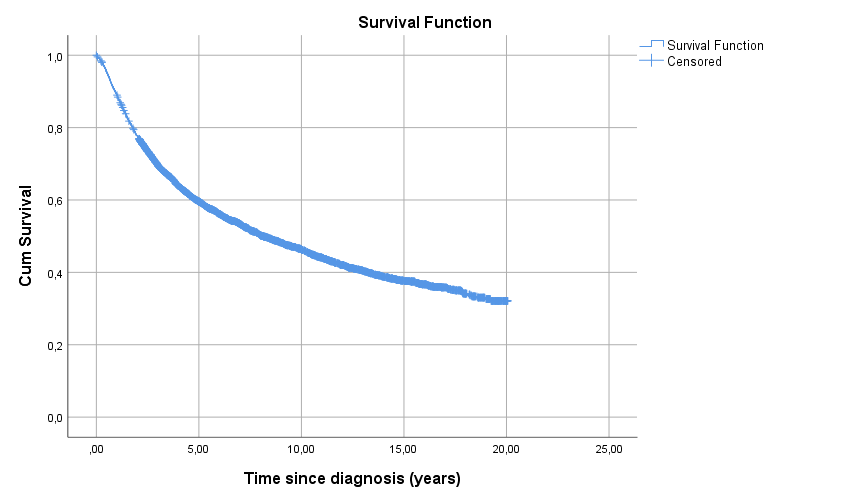


Figure 1: Kaplan-Meier survival curve for grade II and III resected STS in the Netherlands between 2000 and 2017


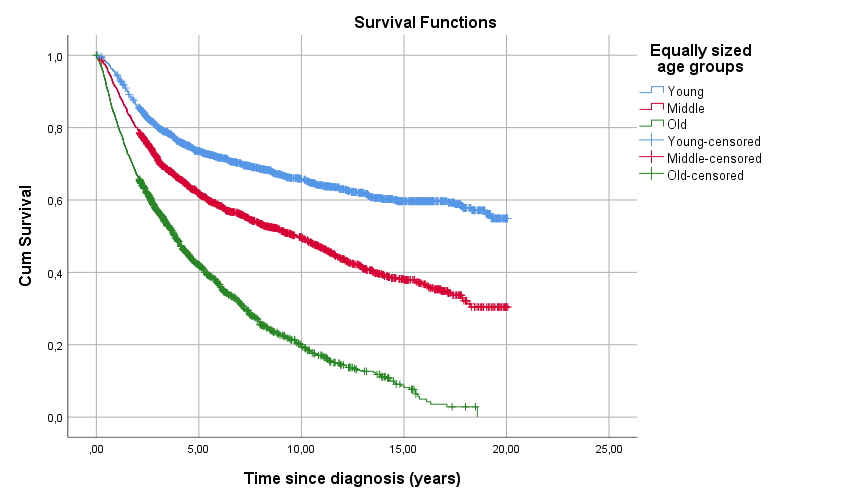


Figure 2: Kaplan-Meier survival curves for grade II and III resected STS in the Netherlands between 2000 and 2017, by equally sized age groups

Table 1: OS at 1, 2, 5 and 10 years along with 95% confidence interval for grade II and III resected STS in the Netherlands between 2000 and 2017, by equally sized age groups

| Equally sized age groups | **1 year OS** | **2 year OS** | **5 year OS** | **10 year OS** |
| --- | --- | --- | --- | --- |
| ‘Young’ (N=1672) | 94.6% (93.4-95.8) | 85.9% (84.1-87.7) | 73.5% (71.3-75.7) | 65.7% (63.2-68.2) |
| ‘Middle’ (N=1667) | 90.7% (89.3-92.1) | 79.9% (77.9-81.9) | 61.8% (59.4-64.2) | 49.6% (46.9-52.3) |
| ‘Old’ (N=1584) | 81.4% (79.4-83.4) | 66.8% (64.4-69.2) | 42.0% (39.5-44.5) | 19.8% (17.3-22.3) |

*p<0.001*


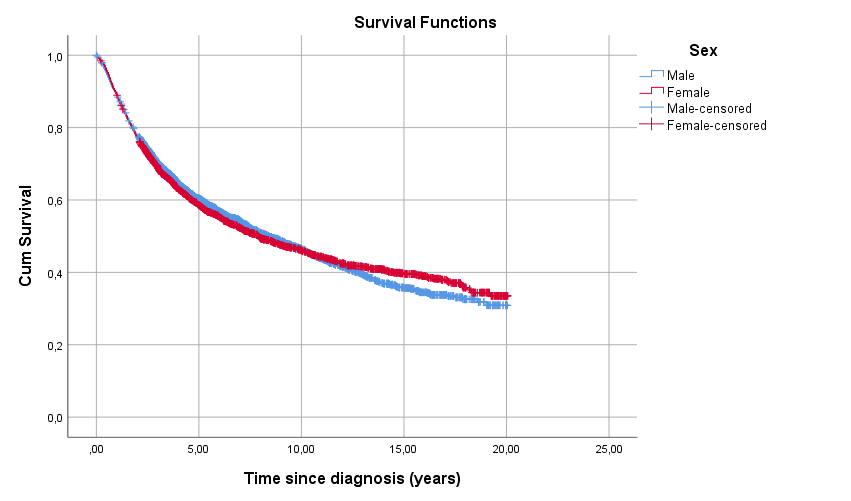


Figure 3: Kaplan-Meier survival curves for grade II and III resected STS in the Netherlands between 2000 and 2017, by sex

Table 2: OS at 1, 2, 5 and 10 years along with 95% confidence interval for grade II and III resected STS in the Netherlands between 2000 and 2017, by sex

| Sex | **1 year OS** | **2 year OS** | **5 year OS** | **10 year OS** |
| --- | --- | --- | --- | --- |
| 1=Male (N=2695) | 89.0% (87.8-90.2) | 78.1% (76.5-79.7) | 60.4% (58.4-62.4) | 46.5% (44.3-48.7) |
| 2=Female (N=2228) | 89.0% (87.6-90.4) | 77.3% (75.5-79.1) | 58.6% (56.4-60.8) | 46.1% (43.7-48.5) |

*p=0.932*


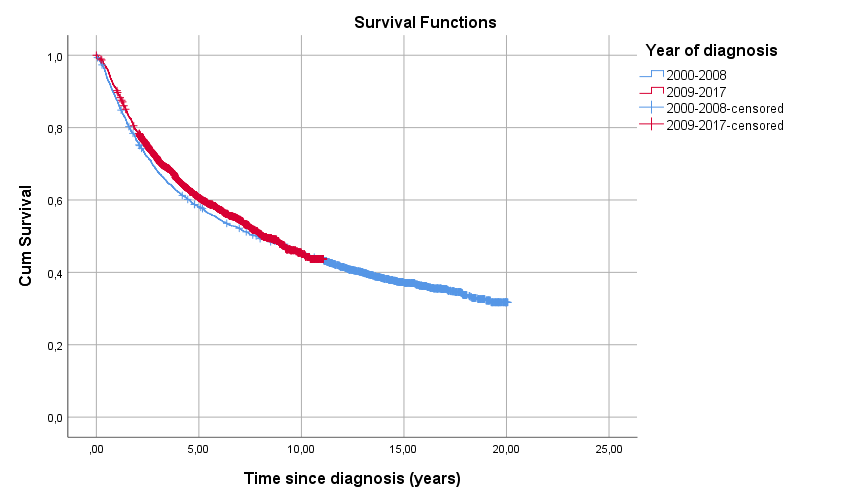


Figure 4: Kaplan-Meier survival curves for grade II and III resected STS in the Netherlands between 2000 and 2017, by year of diagnosis

Table 3: OS at 1, 2, 5 and 10 years along with 95% confidence interval for grade II and III resected STS in the Netherlands between 2000 and 2017, by year of diagnosis

| Year of diagnosis | **1 year OS** | **2 year OS** | **5 year OS** | **10 year OS** |
| --- | --- | --- | --- | --- |
| 2000-2008 (N=2254) | 87.5% (86.1-88.9) | 76.2% (74.4-78.0) | 58.2% (56.2-60.2) | 45.8% (43.6-48.0) |
| 2009-2017 (N=2669) | 90.3% (89.1-91.5) | 79.0% (77.4-80.6) | 60.6% (58.6-62.6) | 45.1% (42.2-48.0) |

*p=0.122*


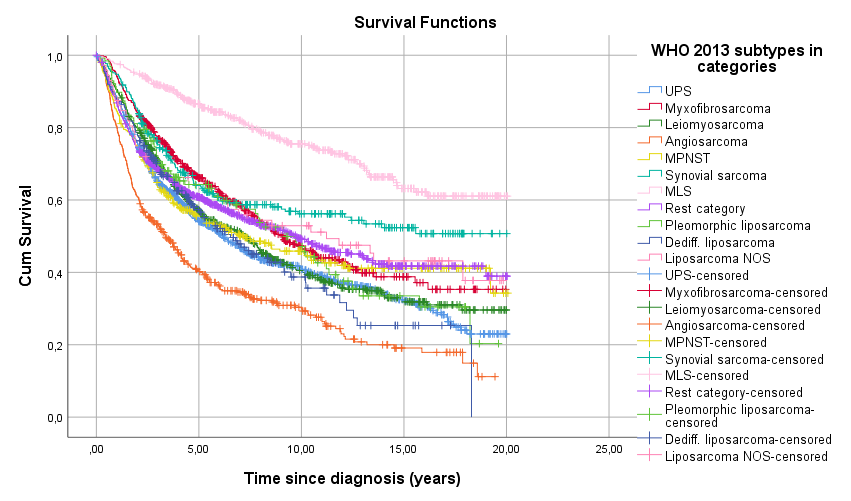


Figure 5: Kaplan-Meier survival curves for grade II and III resected STS in the Netherlands between 2000 and 2017, by histological subtype

Table 4: OS at 1, 2, 5 and 10 years along with 95% confidence interval for grade II and III resected STS in the Netherlands between 2000 and 2017, by histological subtype

| Subtype | **1 year OS** | **2 year OS** | **5 year OS** | **10 year OS** |
| --- | --- | --- | --- | --- |
| UPS (N=935) | 88.2% (86.0-90.4) | 75.9% (73.2-78.6) | 54.2% (50.9-57.5) | 40.8% (37.3-44.3) |
| Myxofibro (N=536) | 94.2% (92.2-96.2) | 84.3% (81.2-87.4) | 66.1% (62.0-70.2) | 47.3% (42.2-52.4) |
| Leiomyo (N=620) | 90.5% (88.1-92.9) | 80.0% (76.9-83.1) | 57.1% (53.2-61.0) | 40.5% (36.2-44.8) |
| Angio (N=419) | 80.4% (76.7-84.1) | 60.9% (56.2-65.7) | 40.2% (35.3-45.1) | 29.8% (24.9-34.7) |
| MPNST (N=313) | 84.6% (80.7-88.5) | 75.0% (70.1-79.9) | 55.6% (49.9-61.3) | 45.9% (39.8-52.0) |
| Synovial (N=267) | 94.8% (92.1-97.5) | 85.4% (81.1-89.7) | 64.0% (58.1-69.9) | 56.2% (49.7-62.7) |
| Myxoid liposarcoma (N=313) | 97.7% (96.3-99.1) | 94.8% (92.6-97.0) | 86.7% (83.4-90.0) | 75.5% (71.0-80.0) |
| Pleomorphic liposarcoma (N=127) | 89.8% (84.4-95.0) | 81.1% (74.2-88.0) | 64.3% (55.7-72.9) | 47.1% (37.1-57.1) |
| Dedifferentiated liposarcoma (N=220) | 87.7% (83.4-92.0) | 78.6% (73.1-84.1) | 57.4% (50.3-64.5) | 38.7% (30.3-47.1) |
| Liposarcoma NOS (N=72) | 87.5% (79.9-95.1) | 74.8% (64.8-84.8) | 66.2% (55.2-77.2) | 52.9% (41.1-64.7) |
| Rest category (N=815) | 86.8% (84.4-89.2) | 74.0% (71.1-76.9) | 60.8% (57.3-64.3) | 49.1% (45.0-53.2) |

*P<0.001*


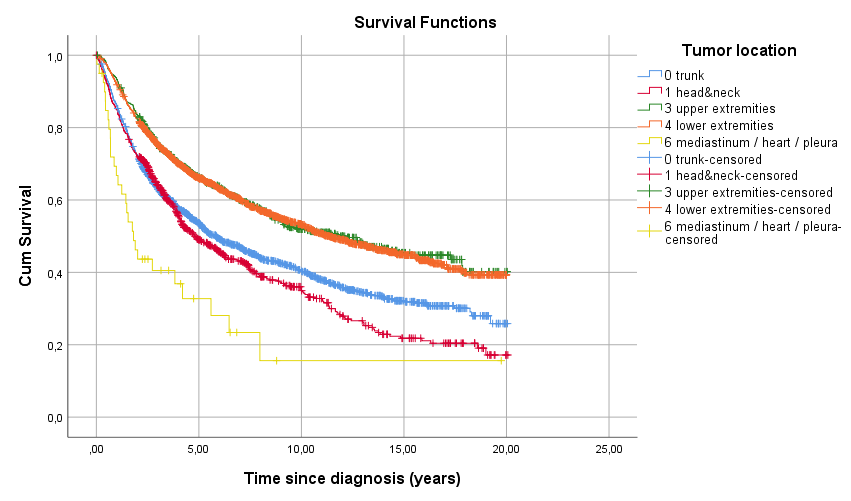


Figure 6: Kaplan-Meier survival curves for grade II and III resected STS in the Netherlands between 2000 and 2017, by tumor location

Table 5: OS at 1, 2, 5 and 10 years along with 95% confidence interval for grade II and III resected STS in the Netherlands between 2000 and 2017, by tumor location

| Tumor location | **1 year OS** | **2 year OS** | **5 year OS** | **10 year OS** |
| --- | --- | --- | --- | --- |
| Trunk (N=1521) | 86.0% (84.2-87.8) | 72.5% (70.3-74.7) | 53.6% (51.1-56.1) | 40.4% (37.7-43.1) |
| Head&neck (N=724) | 84.9% (82.4-87.4) | 72.1% (68.8-75.4) | 49.0% (45.1-52.9) | 34.9% (30.6-39.2) |
| Upper extr. (N=699) | 93.3% (91.5-95.1) | 83.5% (80.8-86.2) | 66.4% (62.9-69.9) | 51.9% (47.8-56.0) |
| Lower extr. (N=1939) | 91.9% (90.7-93.1) | 82.5% (80.7-84.3) | 66.1% (63.9-63.9) | 53.3% (50.8-55.8) |
| Mediastinum, heart, pleura (N=40) | 66.8% (52.1-81.5) | 46.2% (30.5-61.9) | 32.8% (16.9-48.7) | 15.6% (0.0-31.9) |

*P<0.001*


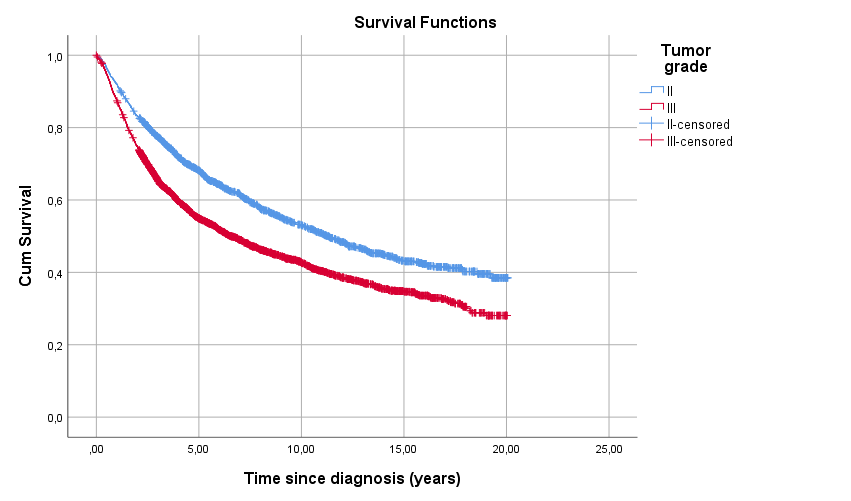


Figure 7: Kaplan-Meier survival curves for grade II and III resected STS in the Netherlands between 2000 and 2017, by grade

Table 6: OS at 1, 2, 5 and 10 years along with 95% confidence interval for grade II and III resected STS in the Netherlands between 2000 and 2017, by grade

| Tumor grade | **1 year OS** | **2 year OS** | **5 year OS** | **10 year OS** |
| --- | --- | --- | --- | --- |
| II (N=1703) | 92.1% (90.7-93.5) | 83.2% (81.4-85.0) | 68.2% (65.8-70.6) | 53.2% (50.5-55.9) |
| III (N=3220) | 87.4% (86.2-88.6) | 74.8% (73.2-76.4) | 54.9% (53.1-56.7) | 42.6% (40.6-44.6) |

*p<0.001*


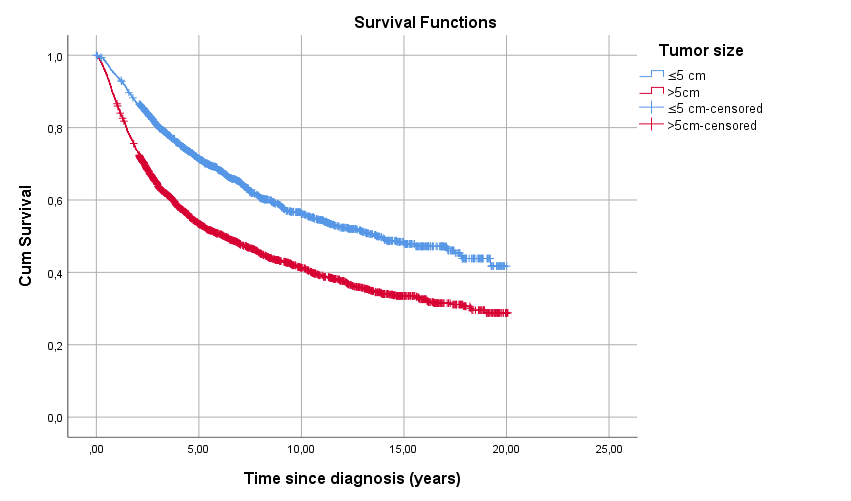


Figure 8: Kaplan-Meier survival curves for grade II and III resected STS in the Netherlands between 2000 and 2017, by tumor size

Table 7: OS at 1, 2, 5 and 10 years along with 95% confidence interval for grade II and III resected STS in the Netherlands between 2000 and 2017, by tumor size

| Tumor size | **1 year OS** | **2 year OS** | **5 year OS** | **10 year OS** |
| --- | --- | --- | --- | --- |
| ≤5 cm (N=1651) | 94.2% (93.0-95.4) | 86.7% (85.1-88.3) | 71.4% (69.0-73.8) | 56.4% (53.7-59.1) |
| >5 cm (N=2194) | 86.6% (85.2-88.0) | 73.7% (71.9-75.5) | 53.4% (51.2-55.6) | 41.3% (38.9-43.7) |

*p<0.001*


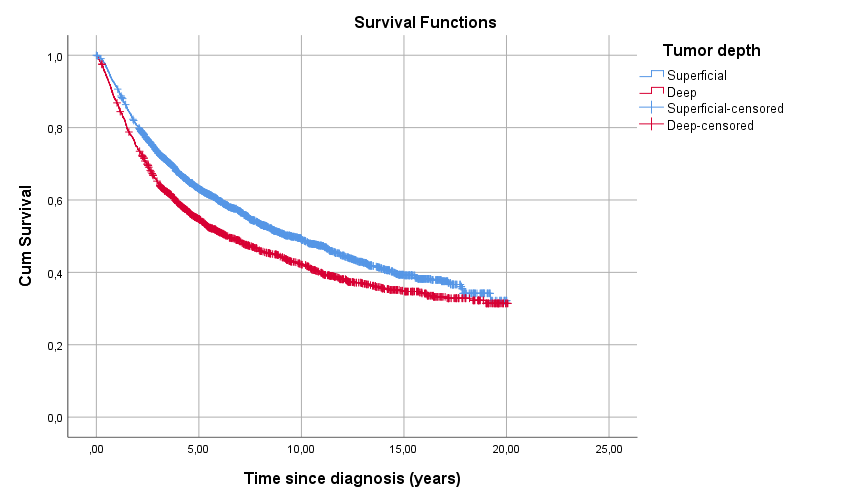
Figure 9: Kaplan-Meier survival curves for grade II and III resected STS in the Netherlands between 2000 and 2017, by tumor depth

Table 8: OS at 1, 2, 5 and 10 years along with 95% confidence interval for grade II and III resected STS in the Netherlands between 2000 and 2017, by tumor depth

| Tumor depth | **1 year OS** | **2 year OS** | **5 year OS** | **10 year OS** |
| --- | --- | --- | --- | --- |
| Superficial (N=2531) | 91.2% (90.0-92.4) | 80.5% (78.9-82.1) | 63.1% (61.1-65.1) | 49.1% (46.9-51.3) |
| Deep (N=1643) | 86.8% (85.2-88.4) | 74.6% (72.4-76.8) | 54.8% (52.3-57.3) | 42.2% (39.7-44.7) |

*p<0.001*


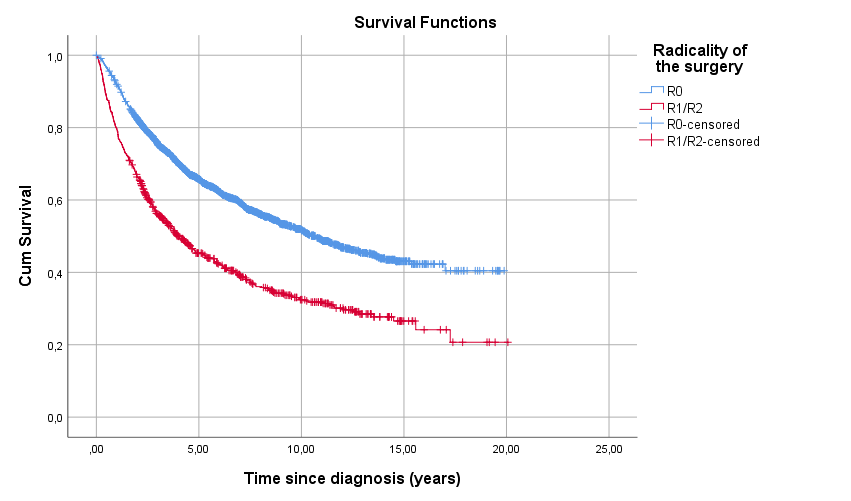


Figure 10: Kaplan-Meier survival curves for grade II and III resected STS in the Netherlands between 2000 and 2017, by radicality of the surgery

Table 9: OS at 1, 2, 5 and 10 years along with 95% confidence interval for grade II and III resected STS in the Netherlands between 2000 and 2017, by radicality of the surgery

| Radicality of the surgery | **1 year OS** | **2 year OS** | **5 year OS** | **10 year OS** |
| --- | --- | --- | --- | --- |
| R0  (N=2558) | 91.8% (90.8-92.8) | 82.4% (80.8-84.0) | 65.8% (63.8-67.8) | 51.8% (49.4-54.2) |
| R1/R2 (N=720) | 79.6% (76.7-82.5) | 66.4% (62.9-69.9) | 45.3% (41.6-49.0) | 32.4% (28.5-36.3) |

*p<0.001*


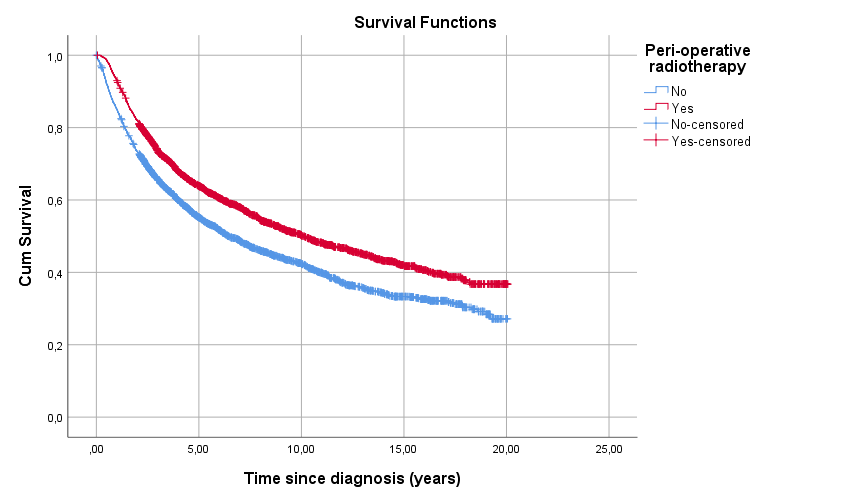


Figure 11: Kaplan-Meier survival curves for grade II and III resected STS in the Netherlands between 2000 and 2017, by peri-operative radiotherapy

Table 10: OS at 1, 2, 5 and 10 years along with 95% confidence interval for grade II and III resected STS in the Netherlands between 2000 and 2017, by peri-operative radiotherapy

| Peri-operative radiotherapy | **1 year OS** | **2 year OS** | **5 year OS** | **10 year OS** |
| --- | --- | --- | --- | --- |
| No (N=2459) | 85.1% (83.7-86.5) | 73.4% (71.6-75.2) | 55.2% (53.2-57.2) | 42.3% (40.1-44.5) |
| Yes (N=2464) | 93.0% (92.0-94.0) | 82.0% (80.4-83.6) | 63.9% (61.9-65.9) | 50.3% (48.1-52.5) |

*p<0.001*


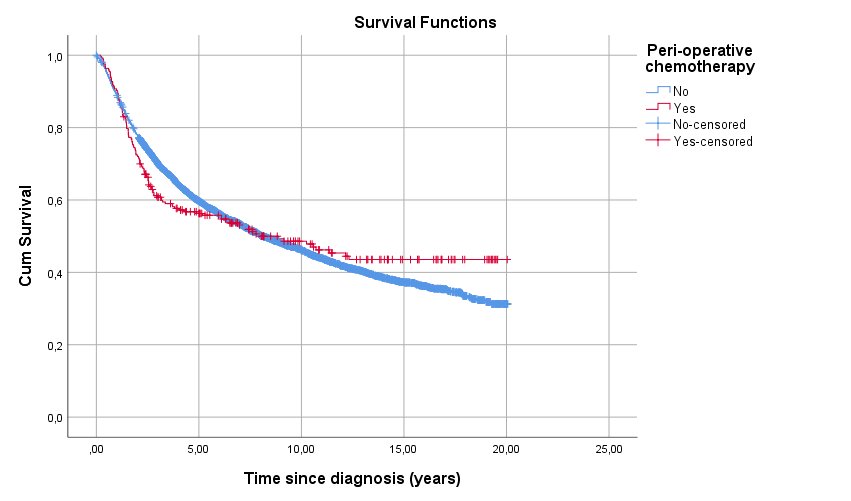


Figure 12: Kaplan-Meier survival curves for grade II and III resected STS in the Netherlands between 2000 and 2017, by peri-operative chemotherapy

Table 11: OS at 1, 2, 5 and 10 years along with 95% confidence interval for grade II and III resected STS in the Netherlands between 2000 and 2017, by peri-operative chemotherapy

| Peri-operative chemotherapy | **1 year OS** | **2 year OS** | **5 year OS** | **10 year OS** |
| --- | --- | --- | --- | --- |
| No (N=4676) | 89.0% (88.0-90.0) | 78.0% (76.8-79.2) | 59.7% (58.3-61.1) | 46.2% (44.6-47.8) |
| Yes (N=247) | 90.3% (86.6-94.0) | 72.4% (66.9-77.9) | 56.3% (50.0-62.6) | 48.6% (41.9-55.3) |

*p=0.859*

Figure 13: Percentage of patients receiving peri-operative chemotherapy per subtype for grade II and III resected STS in the Netherlands between 2000 and 2017

|  | No TT | Post-op TT | Pre-op TT | Post-op & pre-op TT |
| --- | --- | --- | --- | --- |
| 2000-2002 | 625 | 0 | 0 | 0 |
| 2003-2005 | 716 | 0 | 0 | 0 |
| 2006-2008 | 789 | 0 | 2 | 1 |
| 2009-2011 | 770 | 0 | 2 | 0 |
| 2012-2014 | 843 | 0 | 6 | 0 |
| 2015-2017 | 942 | 0 | 10 | 1 |

Table 12: The use of peri-operative targeted therapy for grade II and III resected STS in the Netherlands between 2000 and 2017
